# Embryonic Environmental Niche Reprograms Somatic Cells to Express Pluripotency Markers and Participate in Adult Chimaeras

**DOI:** 10.3390/cells10030490

**Published:** 2021-02-25

**Authors:** Krystyna Żyżyńska-Galeńska, Agnieszka Bernat, Anna Piliszek, Jolanta Karasiewicz, Ewa Szablisty, Mariusz Sacharczuk, Marta Brewińska-Olchowik, Michał Bochenek, Joanna Grabarek, Jacek Andrzej Modliński

**Affiliations:** 1 Department of Experimental Embryology, Institute of Genetics and Animal Biotechnology, Polish Academy of Sciences, Jastrzębiec, 05-552 Magdalenka, Poland; a.bernat@igbzpan.pl (A.B.); a.piliszek@igbzpan.pl (A.P.); j.karasiewicz@op.pl (J.K.); E.Szablisty@igbzpan.pl (E.S.); j.a.modlinski@igbzpan.pl (J.A.M.); 2 Laboratory of Molecular Diagnostics, Department of Biotechnology, Intercollegiate Faculty of Biotechnology, University of Gdańsk & Medical University of Gdańsk, 80-307 Gdańsk, Poland; 3 Department of Experimental Genomics, Institute of Genetics and Animal Biotechnology, Polish Academy of Sciences, Jastrzębiec, 05-552 Magdalenka, Poland; m.sacharczuk@ighz.pl; 4 Centre for Preclinical Research and Technology, Department of Pharmacodynamics, Faculty of Pharmacy with the Laboratory Medicine Division, Medical University of Warsaw, 02-097 Warsaw, Poland; 5 Laboratory of Cytometry, Nencki Institute of Experimental Biology, 02-093 Warsaw, Poland; m.brewinska-olchowik@nencki.edu.pl; 6 Department of Reproductive Biotechnology and Cryoconservation, National Research Institute of Animal Production, 32-083 Balice, Poland; michal.bochenek@uj.edu.pl; 7 Faculty of Biology, Medicine and Health, Division of Developmental Biology & Medicine, University of Manchester, Manchester, M13 9PT, UK; joanna.grabarek@cruk.manchester.ac.uk

**Keywords:** reprogramming, embryonic niche, chimaera, plasticity

## Abstract

The phenomenon of the reprogramming of terminally differentiated cells can be achieved by various means, like somatic cell nuclear transfer, cell fusion with a pluripotent cell, or the introduction of pluripotency genes. Here, we present the evidence that somatic cells can attain the expression of pluripotency markers after their introduction into early embryos. Mouse embryonic fibroblasts introduced between blastomeres of cleaving embryos, within two days of in vitro culture, express transcription factors specific to blastocyst lineages, including pluripotency factors. Analysis of donor tissue marker DNA has revealed that the progeny of introduced cells are found in somatic tissues of foetuses and adult chimaeras, providing evidence for cell reprogramming. Analysis of ploidy has shown that in the chimaeras, the progeny of introduced cells are either diploid or tetraploid, the latter indicating cell fusion. The presence of donor DNA in diploid cells from chimaeric embryos proved that the non-fused progeny of introduced fibroblasts persisted in chimaeras, which is evidence of reprogramming by embryonic niche. When adult somatic (cumulus) cells were introduced into early cleavage embryos, the extent of integration was limited and only cell fusion-mediated reprogramming was observed. These results show that both cell fusion and cell interactions with the embryonic niche reprogrammed somatic cells towards pluripotency.

## 1. Introduction

For a long time the process of differentiation was considered irreversible [1,2,3,4,5], however, subsequent studies have shown that it is possible to reverse the process and reprogram cells into a pluripotent state typical for early embryos [6,7,8]. The pluripotency is defined as the ability of a cell to contribute to all embryonic lineages, including the germline. The first reprogramming experiments concerned the reprogramming of cell nuclei and were carried out by [9] in amphibians. They showed that nuclei from blastula cells of the northern leopard frog (Rana pipiens) introduced to enucleated egg cells can support normal development until the tadpole stage [10]. Further experiments on Xenopus laevis demonstrated full reprogramming of differentiated cells’ nuclei from tadpole intestine epithelium in the environment of enucleated oocytes which, after reconstruction with these nuclei, gave rise to a normal frog [11,12].

Reprogramming of somatic cell nuclei by somatic cell nuclear transfer (SCNT) resulted in cloning Dolly the lamb [7]. Over the past two decades, successful cloning of several domestic and wild mammalian species has been achieved (reviewed by [13]).

When applying the SCNT method, the nucleus of a cell to be cloned is introduced to the cytoplasm of an enucleated oocyte. Cell reprogramming and retaining pluripotency is achieved by nucleo-cytoplasmic interactions. The fusion of somatic cells with pluripotent embryonic stem (ES) cells, embryonal carcinoma (EC) cells or neural stem cells (NSCs) [14,15,16] is another method of reprogramming somatic cells by nucleo-cytoplasmic interactions, but the utility of this method is limited as the resultant cells are tetraploid. Cells subjected to the influence of cell extracts may also reprogram to a pluripotent state [17]. However, complex mixtures of known and undefined factors from oocytes or pluripotent cells triggering reprogramming make mechanistic studies more difficult. These barriers may be overcome by a ground-breaking methods in which pluripotency genes are introduced into mammalian somatic cells. When *Oct-4, Sox2, c-Myc* and *Klf4* or *Oct-4, Sox2, Nanog* and *Lin28* were overexpressed in embryonic or adult fibroblasts, the terminal state of differentiation was reversed and led to the derivation of induced pluripotent stem cells (iPSCs) [8,18,19,20,21]. Here, we explore reprogramming of whole donor cells in the environment of a preimplantation mouse embryo. After microsurgical introduction into blastocysts or morula-stage embryos, mouse ES (mES) cells can differentiate into all tissues of the developing foetus, including the germline [22,23]. It has been proved that mES cells alone can support full-term development, either by tetraploid complementation [24] or by inner cell mass (ICM) replacement [25]. Embryo-derived sheep cells, originating from cultured embryonic discs, also retain pluripotency, as after their introduction into host blastocysts, overt chimaeric lambs were obtained [25,26].

It is noteworthy that mES cells have been derived from preimplantation embryos (reviewed in [13]), and as such they relatively easily retain pluripotency upon reintroduction into a blastocyst. On the other hand, [27] have shown that somatic, differentiated hematopoietic cells introduced into the blastocyst cavity may continue to develop and produce blood cells in developing chimaeras. 

We have previously shown that mouse embryonic fibroblasts (MEFs), as well as ovine foetal fibroblasts, introduced into early cleaving embryos, are able to contribute to embryonic and post-natal development in mice and sheep [26,28].

Here, we investigated whether the reprogramming of MEFs and adult cumulus cells can be induced by exposure to a permissive environment of the early mouse embryo, which could be defined as an embryonic niche.

## 2. Materials and Methods

### 2.1. Experimental Outline

Fluorescently or genetically labelled MEFs were introduced into E2.5 (embryonic day 2.5) 8-16-cell recipient embryos and then cultured for 48h until the blastocyst stage. Embryos were divided into three experimental groups. Group 1embryos were fixed and labelled by immunofluorescence for markers of blastocyst lineages. Group 2 and 3 embryos (embryos with integrated cells visible under a confocal or fluorescence microscope) were transferred into pseudo-pregnant females and dissected at E10.5–13.5 (Group 2) or left until birth (Group 3).

Group 1: Blastocyst-stage embryos with fluorescent MEFs were firstly photographed live under the confocal microscope or fluorescence microscope. Localisations of introduced MEF cells were specified and assigned to blastocyst lineages: trophectoderm (TE), primitive endoderm (PrE) or epiblast (EPI). Secondly blastocysts were fixed and stained with antibodies of TE, PrE and EPI markers.

Group 2 and 3: Samples from foetuses and from born animals were analysed for the presence of markers of introduced cells (fluorescent or genetic) and for the ploidy of their progeny.

**Figure 1:**
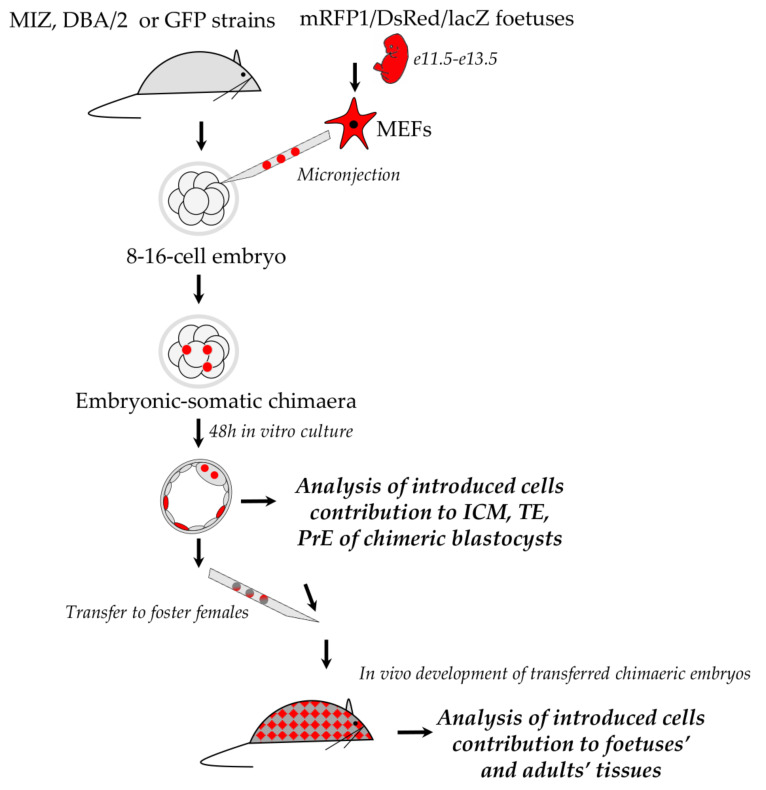
Experimental outline. Figure shows general scheme of experiments performed in this publication.

The schematic representation of the experimental outline is presented in Figure 1.

### 2.2. Animals

Recipient embryos for preimplantation studies (Group 1) were obtained from females of Pdgfra^H2B-GFP^ [29], CAG:GPI-GFP [30], CAG::H2B-EGFP [31] and wild-type mice of mixed background. Recipient embryos for group 2 and 3 (postimplantation studies) were obtained from inbred DBA/2 or MIZ females aged 2–3 months, mated to 3- to 10-month-old males of the same breed.

MEFs of 3 different genetic backgrounds were used. Females of CAG::mRFP1 [32] strains were mated with males of the same strain or MIZ (used for pre- and postimplantation studies). Females of OCT4-GFP-ires-Puromycin (OCT4-GiP) [33,34] were mated with B6.Cg-Tg(CAG-Ds RED*MST)1Nagy/J males (JAX Mice, [35]) (used only for preimplantation studies). Females of ROSA26-lacZ (C57BL10 strain carrying *lacZ* transgene) or CBA/H-T6 strains were mated with males of the other strain to obtain F1 (ROSA26-lacZxCBA/H-T6) foetuses (used only for postimplantation studies).

For embryo transfer, F1 (C57BL10xCBA/H) or F1 (CBA/HxC57BL10) females mated to vasectomised F1 males were used as surrogates. In cases where the DBA/2 embryo strain was used, MIZ females were used as surrogates instead.

### 2.3. Experimental Procedures

#### 2.3.1. Embryo Collection

Experiments were performed in the Department of Experimental Embryology in the Institute of Genetics and Animal Breeding, Polish Academy of Sciences with permission no. 58/2006 of the Third Local Ethical Committee on Animal Experimentation in Warsaw and in a designated facility of the University of Manchester, in accordance with the European Community regulation 86/609. Mice were kept under a 12 h day cycle starting at 06:00 h.

To obtain eight-cell embryos, females were caged with males in the evening and inspected for vaginal plugs the next morning. Those females that mated were sacrificed by cervical dislocation 48–50 h later. Oviducts were excised into M2 manipulation medium (HEPES-buffered M16) and their contents—8-16-cell embryos—were flushed with the same medium using a pipette introduced into the infundibulum.

#### 2.3.2. Mouse Embryonic Fibroblast Isolation 

MEFs were isolated from foetuses from crosses described above. At E11.5–13.5, females were sacrificed by cervical dislocation. MEFs were derived as described previously [28]. Briefly, foetuses were dissected out of their foetal membranes, decapitated and eviscerated. All organs and the head were removed (including gonads) from foetuses and the remaining body shell was cut into small pieces, trypsinised (0.25% trypsin/EDTA for 30 min at 37 °C) and cultured in DMEM (Sigma, St. Louis, MO, USA). Before manipulations, MEFs were trypsinised, then suspended in M2 medium and kept at 4 °C until manipulation (usually one to five hours). 

#### 2.3.3. Adult Somatic Cells Isolation

To separate cumulus cells from ovulated oocytes, cumulus–oocyte complexes were washed in hyaluronidase (0.1%, Sigma, St. Louis, MO, USA) [36]. Next, single cumulus cells were washed and resuspended in M2 medium and kept at 4 °C until manipulation (usually one to five hours).

#### 2.3.4. Embryo Manipulation

Before manipulation, the embryos were incubated (20–60 min) in (1) M2 containing cytochalasin D (CD, Sigma, St. Louis, MO, USA, 1 μg/mL), at 37 °C on a warm plate or (2) in M2 without Ca^2+^ and Mg^2+^ to induce decompaction of embryos. Five to seven embryos were placed in a drop of M2 medium with CD or M2 without Ca^2+^ and Mg^2+^ under paraffin oil in the manipulation chamber. MEFs were kept suspended in M2 at 4 °C and added to the manipulation chamber before each manipulation series. Manipulations were performed on a warm microscope stage at 33–35 °C, as described by [37] under a Fluovert (Leitz, Wetzlar, Germany) inverted microscope, with differential interference Nomarski contrast (DIC). A mechanical Leitz (Wetzlar, Germany) manipulator, micropumps: CellTram Vario, Eppendorf (Hamburg, Germany), connected with an injection pipette, and CellTram Air, Eppendorf (Hamburg, Germany), connected with holding pipette, were used. Both holding and injection pipettes were prepared from thin-walled borosilicate capillaries of external diameter 1 mm, made of silica glass (GC 100T-15, Harvard Apparatus Ltd; Edenbridge, Kent, UK). Three to four MEFs or cumulus cells were introduced into the centre of each eight-cell embryo.

#### 2.3.5. Embryo Culture Conditions

After manipulation, embryos were rinsed thoroughly with M2 without CD and placed in drops of KSOM medium (Specialty Media, Phillipsburg, NJ, USA) under paraffin oil (Sigma, St. Louis, MO, USA) in Petri dishes (Corning, NY, USA) and cultured at 37 °C, in an atmosphere of 5% CO_2_ for 48 h. 

#### 2.3.6. Embryo Transfer

Foster females were anaesthetised by i.p. injections of 0.01 mL/g body weight of 0.25% solution of Vetbutal (Biovet, Pulawy, Poland) in PBS or by xylazine/ketamine mixture (100 mg/kg ketamine, Biovet, 5 mg/kg xylazine, Sigma, St. Louis, MO, USA; 0.1mL/20g mouse, [38]). The embryos (5–10 per transfer) were transferred to the uteri of recipients during the third day of pseudopregnancy.

#### 2.3.7. Obtaining Chimaeric Foetuses and Animals

Pregnant females were sacrificed at day 8 to 10 after the transfer (i.e., E10.5–13.5). Dissected foetuses were observed under a stereomicroscope to evaluate their developmental stage (according to [39]). Samples of the amnion, yolk sac and the embryo proper were collected for the analysis of ploidy and DNA analysis by FACS.

Group 3 of recipients was left to develop to term. The majority of the born animals was sacrificed by cervical dislocation as adults (at 1–2 or 8–9 months of age). Samples of brain, gonad, heart, intestine, kidney, liver, lung, muscle, skin, spleen and bone marrow (and in one case of the tumour) were used for DNA isolation and further analysis. 

#### 2.3.8. Immunostaining 

The zona pellucida was removed using acid Tyrode’s solution (Sigma, St. Louis, MO, USA). Embryos were fixed in 4% paraformaldehyde (PFA) in PBS with 0.1% Tween 20 (Sigma, St. Louis, MO, USA) and 0.01% Triton X-100 (Sigma St. Louis, MO, USA) overnight at 4 °C, permeabilised in 0.55% Triton X-100 in PBS for 15 minutes and blocked in 10% foetal bovine serum in PBS for 1 hour. The following primary antibodies and dilutions were used: mouse anti-CDX2 (BioGenex, Fremont, CA, USA) 1:1, goat anti-GATA4 (C-20, Santa Cruz, Dallas, TX, USA) 1:100, rabbit anti-NANOG (Cosmo Bio, Carlsbad, CA, USA) 1:300. Secondary antibodies were: Alexa Fluor at 1:500 (donkey anti-goat Alexa 488, donkey anti-rabbit Alexa 568, donkey anti-mouse Alexa 568, donkey anti-rabbit Alexa 647, donkey anti-rat Alexa 633, Invitrogen, Thermofisher, Grand Island, NY, USA). DNA was visualised using Hoechst 33342 staining (5 μg/mL, Molecular Probes, Thermofisher, Grand Island, NY, USA).

### 2.4. Analyses of Samples 

#### 2.4.1. Image Acquisition, Processing and Analysis

Laser scanning live confocal images were acquired using an Olympus inverted confocal microscope (Fluoview FV1000, Tokyo, Japan) with Olympus Fluoview v2.1 software or a Leica inverted SP5 confocal microscope with Leica LAS software. Immunostained embryos were mounted in Vectashield (Vector Laboratories, Burlingame, CA, USA) on a glass-bottom dish and visualised using: (1) an Olympus inverted confocal microscope (Fluoview FV1000) with Olympus Fluoview v2.1 software or (2) a Leica inverted SP5 confocal microscope with Leica LAS software (Wetzlar, Germany) or (3) a Nikon A1R confocal microscope (Tokyo, Japan). Optical section thickness ranged from 1 μm to 4 μm.

Analysis of images was performed using IMARIS (Bitplane AG, Zurich, CH-8048 Switzerland ), and ImageJ (NIH, Bethesda, MD, USA). The number of nuclei identified by the software was confirmed manually. 

#### 2.4.2. Staging of Foetuses

Foetuses collected for analyses were assessed according to [39]. Embryos collected at E10.5–13.5 were divided into categories: normal (not retarded), retarded 0.5–2 days, retarded more than 2 days, usually at egg cylinder stage. In some cases, only remains of degenerating embryos were found, which were included in a separate category: the implantation sites.

#### 2.4.3. Cytofluorometric Evaluation of Ploidy

Samples for cytofluorometric analysis of ploidy were taken from embryos and their foetal membranes, from the bone marrow of newborn mice and tissues of adult animals after homogenisation and trypsinisation. Bone marrow was flushed out from femora using fine syringes. All cell suspensions were rinsed with culture medium, then twice with PBS and then with ice-cold 70% ethanol and were centrifuged 5 min at 1000 r.p.m. between each rinse. Finally, the pellets were resuspended in ice-cold ethanol for fixation and stored at 4 °C. Before cytofluorometric analysis, the samples were rinsed with PBS and stained with Hoechst 33342 (5 μg/mL, Molecular Probes, Thermofisher, Grand Island, NY, USA). 

A BD LSR Fortessa Cell Analyzer flow cytometer (BD Biosciences, San Jose, CA, USA) was used. Chimaeric embryos were analysed for the presence of fluorescent introduced cells and their ploidy.

#### 2.4.4. Cell Sorting (FACS)

Samples from foetuses and foetal membranes were sorted by flow cytometry to separate fractions containing hybrid DNA (4N). Briefly, fixation medium (ethanol) was removed by centrifugation at 200× *g* for 10 min. Pellets were resuspended in 1ml of PBS and stained with Hoechst 33342 (Sigma, St. Louis, MO, USA ). After 40 min of incubation at 35 °C, the cells were sorted using a MoFlo (Beckman-Coulter, Brea, CA, USA) flow cytometer. Sorting was performed under pressure of 40PSI, PBS as sheath fluid and UV laser power of 150mW. The cells with DNA > 4N (hypertetraploid) were sorted into 15 mL conical tubes. The collecting tubes were prefilled with 0.5 mL of PBS to avoid drying of the first sorted microdroplets. Sorted samples were centrifuged at 200× *g* for 10 min to concentrate the cells and frozen at −20 °C.

#### 2.4.5. Samples for DNA Analysis and DNA Isolation

Pieces of a few cubic millimetres in size were excised from embryos and their membranes and from tissues of adult mice and frozen. DNA was isolated from frozen samples using the DNA Blood and Tissue Kit (Qiagen, 40724 Hilden, Germany) following the protocols of the manufacturer.

#### 2.4.6. DNA Genotyping of the *lacZ* Product 

The presence of the transgene was determined by PCR analysis of genomic DNA using *lacZ*-specific primers:

5′-CTGCGCGATCAGTTCACCCGTGCAC-3′ and 5′-TTTACCCCGCTCTGCTACCTGCGCCA-3′.

The PCR reaction was conducted using a PT-200 thermal cycler (MJ Research, USA) in a total volume of 25 μL that included: 100 ng genomic DNA; 1× reaction buffer (10 mM Tris-HCl pH 8.3, 1.5 mM MgCl, 50 mM KCl); 20 pmol of each primer; 200 μmol of each 2′-deoxyribonucleotide 5′-triphosphate (dNTP); 0.1% DMSO and a 0.5 unit of DNA Taq polymerase (all reagents from Polgen, Poland). The following conditions for the reaction were applied: 3.5 min at 94 °C, followed by 32 amplification cycles of 30 s, at 94 °C, 45 s at 62 °C or 64 °C, 90 s at 72 °C and the final elongation for 10 min at 72 °C. PCR products were then loaded onto a 1% agarose gel with 0.5 μg/mL ethidium bromide (AppliChem, Germany). Horizontal electrophoresis was carried out in LKB-GNA 200 apparatus (Pharmacia, Pfizer, New York, NY 10017) at 100 mA/cm and 120 V for 1 h. PCR fragments were then visualised with the Molecular Imager FX (Bio-Rad, Hercules, CA, USA).

#### 2.4.7. DNA Genotyping of Microsatellites 

Microsatellite markers distributed across autosomes were typed using a polymerase chain reaction (PCR) protocol optimised in the laboratory for each microsatellite. All primer sets were originally designed by the Whitehead Institute/MIT Centre for Genome Research, based on their screens of polymorphic microsatellite loci in mice. Throughout five microsatellites prescreened for their application for chimaerism detection, the best marker was the *D3Mit200* microsatellite, of which allelic forms differ substantially in length between the donor (ROSA26-lacZ) and recipient (DBA/2) strains.

Forward: 5′-CAACTTCAGTTTCTCATTTGAATTG -3′.

Reverse: 5′-GCAAATGGAAGAGGTTTCTCC -3′. 

Amplified fragments’ lengths were 105-127 bp. The amplified fragment represents the core dinucleotide repeat (TG)n and flanking sequences of the murine locus *D3Mit200* (Whitehead Institute at MIT; Centre for Genome Research 1999). The PCR reaction was carried out in a volume of 8.0 µl comprising 100 ng of template DNA, 2.5 pmol of each primer, 100 µM of each dNTP, 0.5 unit of DNA *Taq* polymerase, 10 mM tris-HCl (pH 8.8), 1.5 mM MgCl_2_, 50 mM KCl and 0.1% Triton X-100. One primer for each locus was labelled with fluorescein (indodicarbo-cyanine, Cy5). The PCR reaction was carried out in a thermal cycler (MJ Research PTC-200, Hampton, NH, USA) as follows: 5 min of denaturation at 94°C, followed by 35 cycles of denaturation at 94 °C for 45 s, annealing at 48–68 °C and a final elongation cycle at 72 °C for 10 min. The fluorescent PCR products were separated on 6% denaturing polyacrylamide gels, using an Automated Laser Fluorescent (ALEexpress) DNA Sequencer. The PCR products were analysed after 5 min of denaturation in a 50% formamide solution containing blue dextran. In each lane, PCR products, differing in size range, were loaded together with a standard size marker. The results were visualised and the genotyping was completed with Allele Links 1.01 software (Thermofisher, Grand Island, NY, USA). After automated allele calling and binning within Allele Links 1.01, individual genotypes were manually inspected before exporting the genotype database to Excel.

#### 2.4.8. Sex Identification in Foetuses 

The PCR-based assay to evaluate the presence of the *Sry* gene was designed from Gene Bank sequence MGI:681. The PCR primers used to amplify a 380 bp *Sry* product were:

*SRY*: 5’-TCTTAAACTCTGAAGAAGAGAC-3’ and *SRY*: 5’-GTCTTGCCTGTATGTGATGG-3’. 

The PCR reaction was conducted using the same reaction mixture and temperature profiling as for the *lacZ* fragment with a change of the temperature of annealing to 61 °C.

## 3. Results

### 3.1. Preimplantation Development of Presumptive Embryonic–Somatic Chimaeras Obtained by MEF Introduction to recipient morulae 

#### 3.1.1. MEFs Introduced into 8-16-Cell Recipient Embryo Can Integrate with the Embryo within 2 Days of Culture

Our previous studies have shown that MEFs placed between blastomeres of the mouse morula are able to partially integrate with the embryo [28]. Based on this observation, we introduced 3-4 single RFP-positive MEFs (CAG::mRFP1) into wild-type or GFP-positive 8-16-cell embryos (either Pdgfra^H2B-GFP^ or CAG:GPI-GFP). The resulting chimaeras were cultured for 48 h until the blastocyst stage. We observed that out of 572 embryos with introduced cells that developed to a blastocyst blastocyst stage, 38% (220 blastocysts) contained cells inside of the host embryo. 

Next, we asked whether these MEFs (or their progeny) were located in a specific embryonic compartment (TE or ICM). Integrated cells were mostly found in trophectoderm (TE, 56% of the embryos), but also in the inner cell mass (ICM, 23% of the embryos) or both TE and ICM (21% of the embryos). 

#### 3.1.2. Integrated MEFs Express Markers of Three Blastocyst Lineages

MEFs cultured in vitro do not core express pluripotency factors *Oct4* and *Nanog* (Figure S1). We addressed the question whether MEF cells introduced to the cleavage-stage embryo could be reprogrammed during two days of culture to express pluripotency factors or other markers of blastocyst lineages. Based on live confocal images of chimaeric blastocysts after 48h of culture, the localisation of introduced cells within blastocysts (in TE or ICM) was verified. Depending on MEFs’ localisation in blastocysts, in TE, PrE or EPI, embryos were fixed and stained with the corresponding antibody: CDX2 for TE [40,41], GATA4 for PrE [42,43] and NANOG for EPI [44,45].

**Figure 2:**
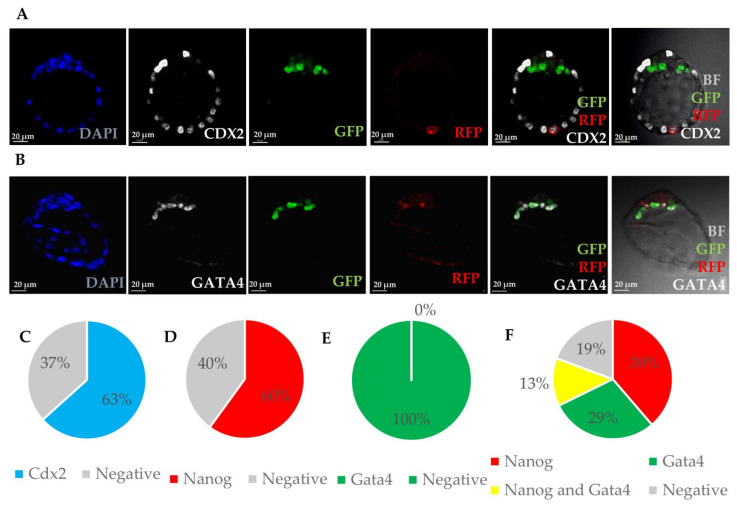
Expression of markers of blastocyst lineages in chimaeric blastocysts. (**A**) Immunofluorescence staining of chimaeric blastocyst. Chimaeric blastocyst with MEF cell expressing RFP (red) incorporated into trophectoderm and expressing CDX2 (white). Recipient embryo is expressing Pdgfra-GFP (green) in PrE cells. (**B**) Immunofluorescence staining of chimaeric blastocyst with MEF cells expressing RFP incorporated into primitive endoderm, 2 cells express Pdgfra-GFP and GATA 4, 1 cell expresses only GATA 4. (**C**) Chart showing percentage of cells stained for CDX2, positive and negative within cells located in TE stained for CDX2. (**D**) Chart showing percentage of cells stained for NANOG, positive and negative within cells located in ICM stained for NANOG (but not GATA4). (**E**) Chart showing percentage of cells stained for GATA4, positive and negative within cells located in ICM stained for GATA4 (but not NANOG). (**F**) Chart showing percentage of cells stained for NANOG and GATA4, expressing each marker within cells located in ICM, stained for NANOG and GATA4.

MEFs’ progeny incorporated into different embryonic compartments were, in the vast majority of the cases, expressing markers of only one lineage (Figure 2). The TE-specific transcription factor CDX2 was expressed in 63% of MEFs’ progeny incorporated into TE (38/60 cells in 24 embryos) (Figure 2A,C, Table S1). In most cases, the localisation of MEFs within ICM was unclear, however, if both markers of EPI and PrE were used, it was possible to confirm their localisation after staining. Thirty-one embryos were stained for markers of both EPI and PrE (NANOG and GATA4, respectively), out of which 68% expressed only one of the markers (Figure 2D–F, Table S1). Only four cells (13%) localised in ICM expressed both NANOG and GATA4. NANOG did not co-localise with CDX2 in any of the six MEF progeny (2 embryos) stained for both factors. 

#### 3.1.3. Introduced MEFs Can Undergo Fusion with Blastomeres within 2 Days of Culture

To verify whether RFP-positive MEFs introduced between blastomeres undergo fusion with the host cells, we used recipient Pdgfra^H2B-GFP^ embryos, where primitive endoderm cells expressed GFP. Analysis of chimaeric embryos showed cells that were GFP and RFP positive, indicating that some of the introduced cells were fused with recipient PrE cells (Figure 2B). 

We hypothesised that the above observations could be the result of cell fusion occurring between the introduced MEFs and host embryonic cells [46,47]. Therefore, to verify the hypothesis of cell fusion, we used transgenic embryos in which all nuclei were fluorescently labelled with H2B-GFP [31] to track the cells originating from the host. In some cases, the expression of epiblast or PrE markers in the donor cells was accompanied by recipient-derived nuclear fluorescence, indicating fusion between cells (42%). 

**Figure 3:**
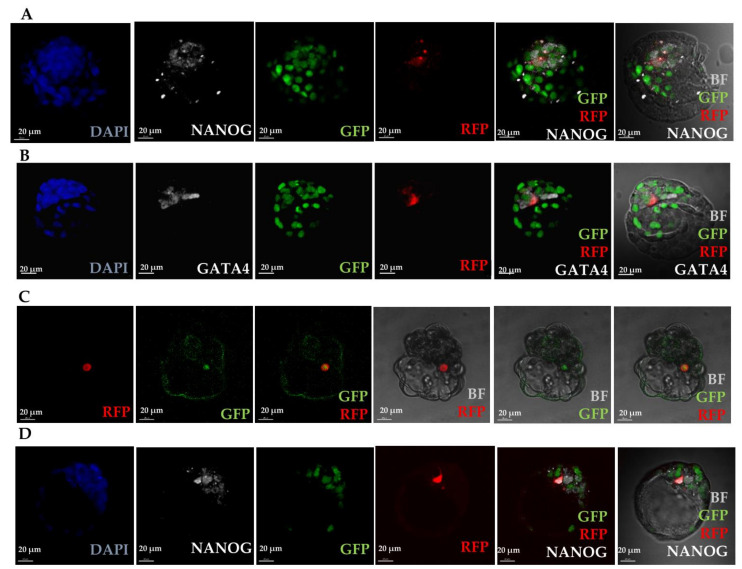
Expression of markers of blastocyst lineages in chimaeric blastocysts. Analysis of fusion of introduced cells and activation of GFP under the promoter of *Oct4*. (**A**) Immunofluorescence staining of chimaeric blastocyst with MEF cell incorporated into epiblast and expressing RFP, GFP and NANOG. Recipient embryo is expressing GFP in all nuclei. Integrated cell expressing RFP and GFP is a product of fusion. (**B**) Immunofluorescence staining of chimaeric blastocyst with MEF cell incorporated into primitive endoderm, expressing RFP and GATA4, but not GFP. Introduced cell is reprogrammed to express GATA4, but is not a product of fusion. (**C**) Live fluorescence imaging of chimaeric blastocyst with MEF cell incorporated into primitive endoderm and expressing RFP and OCT4-GFP (GFP activated under the promoter of *Oct4*). (**D**) Immunofluorescence staining of chimaeric blastocyst with MEF cell incorporated into epiblast and expressing RFP and NANOG. Recipient embryo is expressing GFP in all nuclei. Integrated cell expressing RFP and NANOG, but is not a product of fusion.

However, there were substantial numbers of introduced cells expressing either NANOG (epiblast; 58% of NANOG + cells, n = 19/33, Figure 3A) or GATA4 (PrE; 67% of GATA4+ cells, n = 12/18, Figure 3B) showing no nuclear fluorescence originating from the host embryo. In these numerous cases, reprogramming of introduced somatic cells might have taken place without fusion. 

To confirm reprogramming of introduced cells in the embryonic environment, we used double transgenic donor cells constitutively expressing Ds-Red marker in cytoplasm and conditionally expressing GFP under the promoter of *Oct4(POU5F1)*. A group of 64 embryos was photographed every 12h to observe activation of GFP expression, and in 25 (39%), activation of GFP at various time points was confirmed (Figure S2). In the resulting chimaeras, we found that GFP was expressed in 55% of introduced cells’ progeny that integrated with TE, 20% of cells integrated with epiblast and 70% of cells integrated with PrE (Figure 3C), confirming that OCT4 expression was activated and that at least some cells had undergone reprogramming toward pluripotency.

### 3.2. Postimplantation Development of Presumptive Chimaeras

#### 3.2.1. MEF Progeny Continue Development in Embryonic–Somatic Chimaeras

To verify whether the reprogrammed cells can contribute to both embryonic and extraembryonic lineages during further development, we selected blastocysts carrying introduced MEFs and transferred them to foster mothers. In this set of experiments, we used MEFs of the ROSA26-lacZ strain, carrying genetic marker *LacZ* and microsatellite marker *D3Mit200* that distinguishes introduced cells from the recipient DBA/2 strain. 

**Figure 4:**
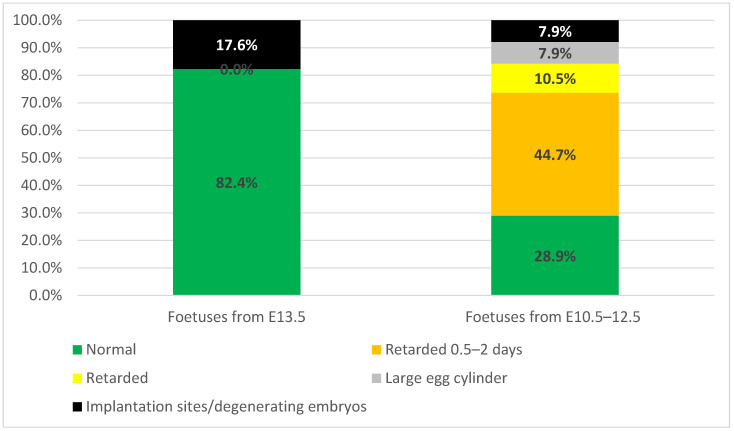
Development of chimaeric foetuses. Chart showing percentage of foetuses with normal and delayed or abnormal development. The number 0.0% in the category “Foetuses from E13.5” reflects the lack of retarded foetuses in this group.

Two hundred and twenty two DBA/2 chimaeric embryos with confirmed MEF contribution were transplanted to the uteri of 29 recipient mothers. Out of these, 62% (n = 18) of females were found pregnant, with a total of 25% (n = 55) implantation sites. The first three autopsies were performed at day 10 after transfer and all of the recovered foetuses (n = 17) were morphologically normal, typical for E13.5. Apart from normal foetuses, three implantation sites (17.6%) with degenerating embryos, and no retarded or abnormal foetuses, were found (Figure 4, Table S2). Therefore, we have chosen an earlier timepoint: 7–9 days after the transfer (E10.5–E12.5) for embryo recovery, to assess the presumed early losses. Normal foetuses comprised 73.7% of implantation sites and the retarded embryos, 18.4%. Some implantation sites were found empty, containing only trophoblastic remnants without the embryo proper, referred to as embryos in the process of resorption. Degenerating egg cylinders and embryos in the process of resorption comprised 7.9% of cases (Figure 4).

**Figure 5:**
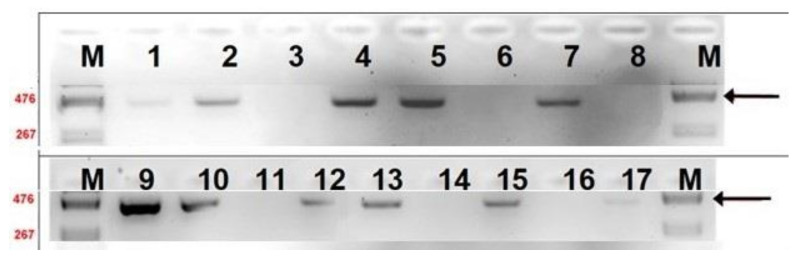
Analysis of sex marker *Sry* in foetuses by PCR reaction. M: DNA size marker 1, 2, 4, 5, 8, 9, 13: normal foetuses; 14, 15: foetuses delayed 0.5–2 days; 3, 10, 12, 16: delayed foetuses; 6, 7, 11, 17: degenerating tissues.

To analyse the sex of recovered foetuses, we used *Sry* marker amplification by PCR (Figure 5, Table S3) for the foetuses recovered at E12.5 (13 foetuses) and E13.5 (four foetuses). In 11 out of 17 samples analysed, *Sry* was present, indicating that 64.7% of the foetuses carried a Y chromosome. Interestingly, whereas 85.7% of normal foetuses were males, in all other groups (from slightly retarded to degenerating foetuses), the male to female ratio was 1:1 (50% *Sry*+). Due to the small number of samples, this result was not statistically significant, *p* > 0.1, however, we see some tendency towards male sex.

#### 3.2.2. Somatic Cells Introduced into Early Embryos are Present in Chimaeric Foetuses and Foetal Membranes

To confirm that introduced cells continue further embryonic development, we analysed samples from 19 implantation sites collected at E12.5 and E13.5 for the *D3Mit200* microsatellite. The analysis revealed specific alleles of the donor strain (ROSA26-lacZ strain): *D3Mit200* microsatellite DNA of 125 or 127 bp long, and recipient strain allele (DBA/2 strain) of 105 bp long. Therefore, we analysed foetuses for the presence of two markers of donor strains: *lacZ* transgene and *D3Mit200* microsatellite. 

**Figure 6:**
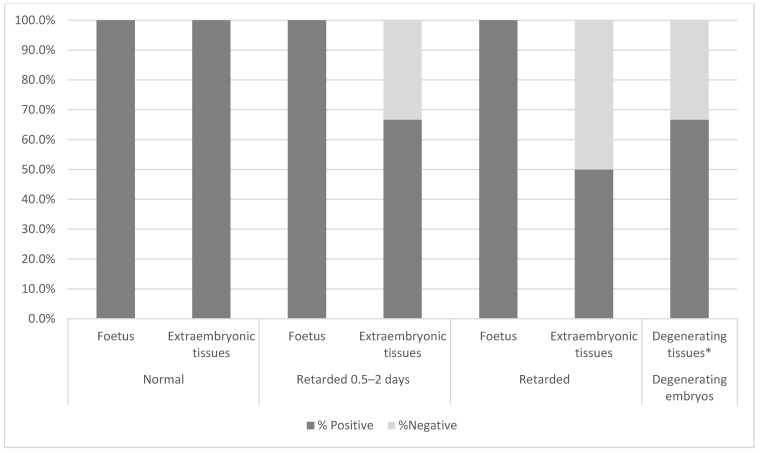
Donor marker expression in foetuses. Chart showing percentage of foetuses expressing donor markers. * all material found in implantation sites possibly containing degenerating embryo.

Sixteen samples from foetuses and fourteen from extraembryonic membranes were isolated from seven normal embryos, three embryos retarded 0.5–2 days, three retarded foetuses and six implantation sites and analysed for *lacZ* DNA and/or *D3Mit200* DNA. Out of 31 samples, 27 (87%) displayed a contribution from one or two donor markers (Figure 6, Table S4). All thirteen normal or retarded embryos tested were positive for either *lacZ* or donor microsatellite allele DNA. Four out of six degenerating or resorbed embryos were donor DNA positive. 

These results show that all normal and retarded foetuses carried donor DNA. This indicates the high efficiency of ICM colonisation by MEFs.

#### 3.2.3. Donor Markers are Found in both Diploid and Tetraploid Cells 

**Figure 7:**
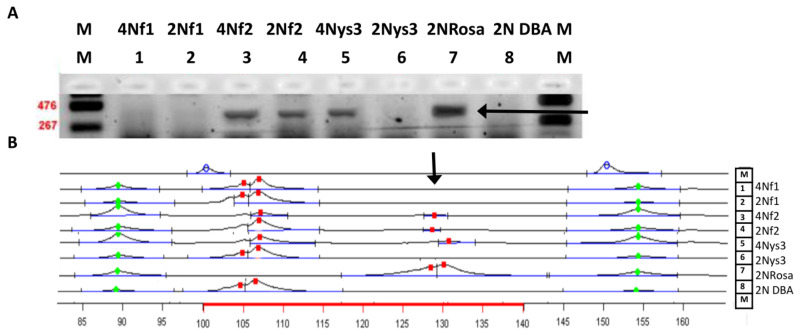
Donor markers in sorted fractions of diploid and tetraploid cells. (**A**) Photography of agarose gel after electrophoresis (**B**) microsatellite marker analysis. M: marker; 1: 4n cells from foetal tissues of foetus 1; 2: 2n cells from the same material; 3: 4n cells from foetal tissues of foetus 2 (both markers present); 4: 2n cells from the same material (both markers present); 5: 4n cells from yolk sac of foetus 3 (both markers present); 6: 2n cells from yolk sac of foetus 3; 7: 2n foetal tissues from ROSA donor (both markers present); 8: 2n foetal tissues from DBA/2 donor; M: marker. Arrows show donor marker occurrence. Red marks indicate the appropriate length of the microsatellite sequences identified in the cells; green marks indicate the appropriate size of the additional flanking length markers.

To address the question of whether donor cells were present as the diploid progeny of introduced MEFs, or as tetraploid progeny of fused donor and recipient cells, we separated diploid and hypertetraploid fractions (i.e., cells that contained more than 4C DNA) by FACS. Samples of three foetuses and their respective yolk sacs at E11.5–E12.5 were analysed for the presence of a donor *lacZ* gene and microsatellite alleles in separated fractions (Figure 7, Table S5). In one E11.5 male foetus, donor *lacZ* and *D3Mit200* were found in both diploid and hypertetraploid fractions (foetus 2 in Figure 7). Both markers were also detected in the fraction of hypertetraploid cells from the yolk sac of another foetus (foetus 3 in Figure 7).

Due to the small size of the selected samples, it was not possible to confirm the presence of donor markers in some foetuses otherwise proven to be chimaeric. Nevertheless, the results showed that the markers of the donor strain may be present in both diploid and tetraploid cells of mid-pregnancy chimaeric foetuses and foetal membranes, indicating that introduced MEFs can contribute both normal and tetraploid cells to chimaeras. 

#### 3.2.4. Fluorescently Labelled Progeny of Introduced Cells Are found in Chimaeric Foetuses and Foetal Membranes and Are Frequently Tetraploid

**Figure 8:**
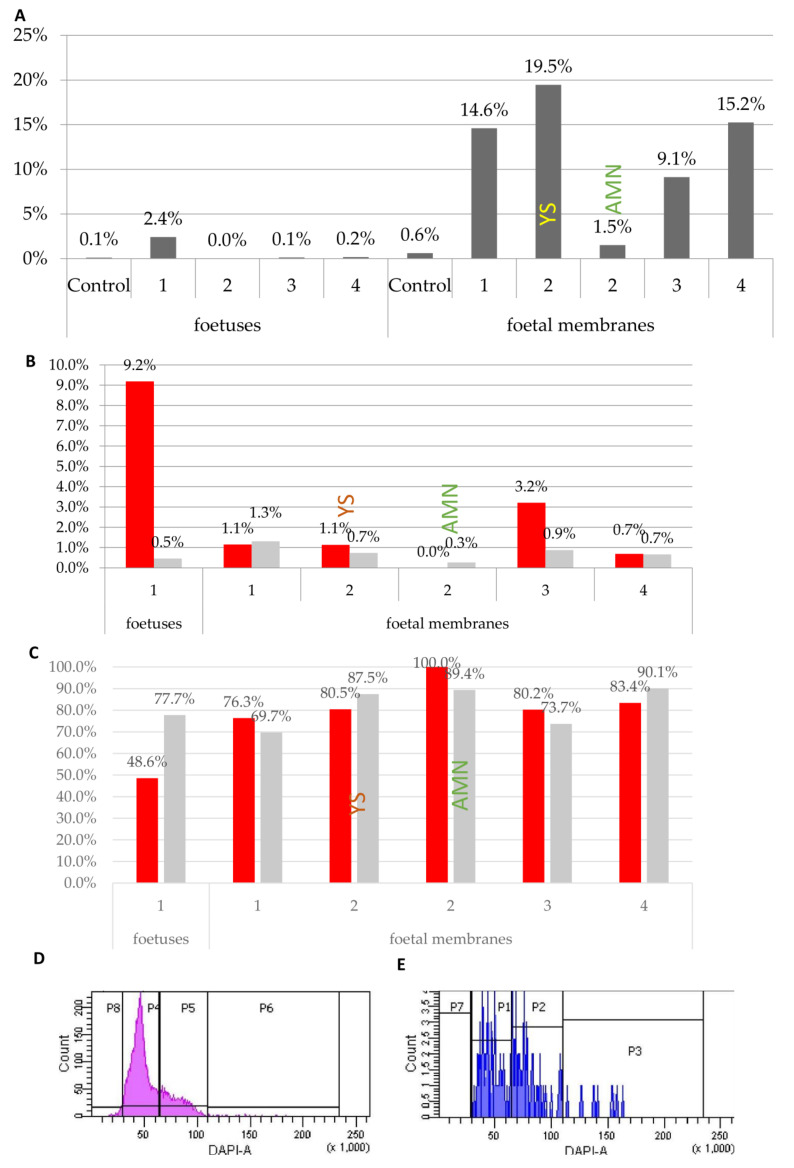
Analysis of donor marker expression in foetuses and foetal membranes. (**A**) Percentage of RFP-expressing cells in foetuses and foetal membranes. Statistically significantly more RFP-positive cells in samples compared to control were found in a chi-square test *p* < 0.01: embryo 1, foetal membranes 1, yolk sac 2, foetal membranes 3, foetal membranes 4. (**B**) Percentage of confirmed tetraploid cells in G2/M cell cycle phase within foetuses and foetal membranes in which a significant number of RFP-expressing cells were found. (**C**) Percentage of diploid cells in G1 cell cycle phase within RFP-expressing and RFP-negative cells in samples, in which RFP-expressing samples were found. (**D**) Cell cycle analysis of RFP-negative cells in foetus 1: P8: <2c DNA, P4: G1 of 2n cells, P5: G2/M of 2n cells and G1 of 4n cells, P6: G2/M of 4n cells. (**E**) Cell cycle analysis of RFP-expressing cells in foetus 1: P7: < 2c DNA, P1: G1 of 2n cells, P2: G2/M of 2n cells and G1 of 4n cells, P3: G2/M of 4n cells.

Previously, genetic markers of donor cells were found both in diploid and tetraploid fractions from chimaeric foetuses. To analyse the ploidy of MEF progeny, we introduced RFP-expressing cells into early cleavage-stage embryos which we transferred into foster mothers. Four presumptive chimaeric foetuses and corresponding foetal membranes were recovered at E12.5 and E13.5 and then analysed by flow cytometry. For the analysis of data, gates for single cells and for tetraploidy were set in relation to control foetuses and foetal membranes separately. In one out of four foetuses and in extraembryonic samples from foetuses 1, 3 and 4 as well as in the yolk sac of foetus 2, a significant number of RFP-positive cells was found (chi-square test, *p* < 0.01) (Figure 8A). Analysis of ploidy confirmed the presence of tetraploid cells in the RFP-positive foetus, and in one of the extraembryonic tissue samples (Figure 8B,E). However, a substantial number of RFP-positive cells were diploid (Figure 8C,E). 

These results confirmed that introduced cells contribute to further development and at least some of them become tetraploid. As the majority of RFP-expressing cells were diploid (Figure 8C), we conclude that both fused and diploid cells can continue development at least until E13.5. 

#### 3.2.5. Pregnancy Rate and Postnatal Survival of Chimaeric Animals

One hundred and forty-two DBA/2 embryos carrying introduced cells were transplanted into 19 recipients. Out of these, 68% (n = 13) of mice were pregnant, giving rise to 42 born animals (29.6% of the total number of embryos; 44.3% of those carried by 13 recipients). The observed mortality among newborns was 9.5%. Out of 37 animals that survived, 15 (40.5%) were females and 22 (59.5%) were males. Four females and one male died later for an unknown reason. One surviving female developed tumours at 3 months of age, and two males at 9 months. One of the latter tumours was subjected to DNA analysis (Table S6).

#### 3.2.6. Donor DNA Markers are Present in Adult Tissues

Tissue samples were collected post mortem from nine presumptively chimaeric animals sacrificed at the age of 9 months, and from one male that died at the age of 1 month for unknown reasons. Samples were collected from up to 10 organs for DNA analysis.

Samples from five animals were tested for the presence of a donor *LacZ* marker and the other five for the presence of a donor DMit200 marker. In nine out of ten animals, chimaerism was confirmed by the presence of a donor marker. *LacZ* was found in all five animals analysed, in four to eight different tissues. The presence of a donor microsatellite marker was confirmed in four out of five animals in one to four organs. Donor microsatellite alleles and *lacZ* were present in derivatives of all three germ layers: ectodermal (brain) tissue as well as tissues originating from the mesoderm (heart, muscle) and endoderm (intestine, lung) (Table S6). 

#### 3.2.7. RFP-Positive Progeny of Introduced MEFs Are Present in Adult Tissues

**Figure 9:**
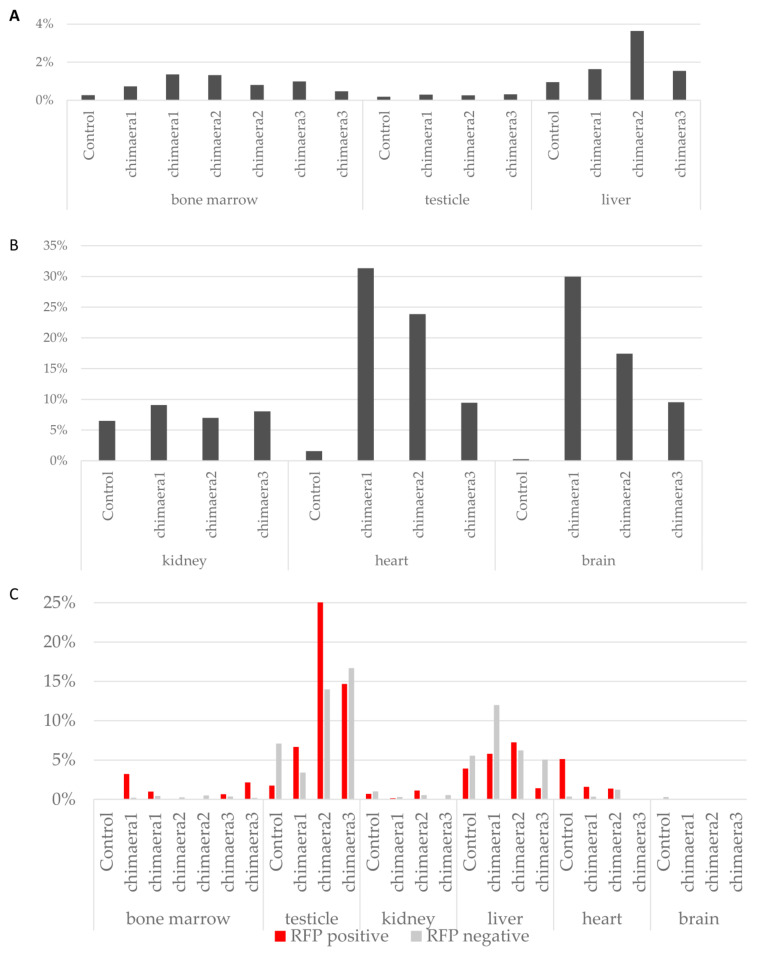
Analysis of donor marker expression in samples from adult animal organs. (**A**), (**B**) Percentage of RFP-expressing cells in organs. Statistically significantly more RFP-positive cells in samples compared to control were found in a chi-square test, *p* < 0.01: bone marrow (all chimaeric samples), testicle (3), kidney (1), kidney (3), liver (all chimaeric samples), heart (all chimaeric samples), brain (all chimaeric samples). (**C**) Tetraploid cells in G2/M cell cycle phase in RFP-positive and RFP-negative samples. Chart shows percentage of tetraploid cells in groups marked as RFP positive (progeny of donor cells) and RFP negative.

To confirm that the progeny of introduced cells can be found in chimaeric animals after birth, DBA/2 embryos with introduced RFP-positive cells were transferred to pseudo-pregnant females and left to develop to term. Twenty-five potentially chimaeric embryos were transferred into uteri of three foster females, out of which two became pregnant. Eight pups were born. Samples from organs of three animals were collected for cytofluorometric analyses. In five tissues from three potentially chimaeric animals, RFP-positive cells were detected by flow cytometry analysis (Figure 9A,B). 

Overall, in tissues of chimaeric pups, red fluorescence was detected in all samples, in 0.5–1.4% of cells, while in control samples (mice of recipient DBA/2 strain), autofluorescence was detected in 0.27% of cells. In all selected and examined tissues, red fluorescent cells were found, albeit at a different percentage. A high percentage of RFP-positive cells, much above the level of the auto-fluorescence signal from control tissues, was detected in the heart (9.4–31.3%) and brain (9.5–29.9%). In some tissues, such as kidney and liver where high natural autofluorescence occurs (6.5% and 1%, respectively), a small proportion of RFP-positive cells was found, an average of 8% for kidney and 2.1% for liver. If injected MEFs do not undergo reprogramming in early embryos, then the MEF progeny would undergo senescence and eventually die off, as this takes place when MEFs are cultured in vitro. If single cells could somehow survive in the developing embryo without senescence and without reprogramming, stalled in their state and not eliminated from rapidly growing foetuses, their number would be extremely small (below the detection threshold), as only three to four cells were introduced into the embryo. Statistically significantly more RFP-positive cells in samples compared to control were found in a chi-square test, *p* < 0.01: bone marrow (all chimaeric samples), testicle (three), kidney (one), kidney (three), liver (all chimaeric samples), heart (all chimaeric samples), brain (all chimaeric samples). 

In testicles, the number of RFP-positive cells was small: 0.3% in all three chimaeric samples and 0.19% in control, and 0.30%, 0.25% and 0.31% in chimaeric samples. The sample from testicle 3 was statistically different from control in a chi-square test at *p* < 0.01. However, the percentage of RFP-positive cells was low and no offspring or MEF origin was obtained.

As our analysis confirmed a significant percentage of tetraploid cells in foetal tissues, we also analysed the possibility of tetraploid cell contribution to adult tissues. The proportion of tetraploid cells was analysed in bone marrow, testis, liver, heart and brain, and all tissues that contained a significant number or RFP-expressing cells. Only in the bone marrow of one chimaera was a large number of tetraploid cells found among RFP-positive cells, and we did not confirm this tendency in other samples (Figure 9C). Likewise, in a previous analysis of ploidy, tetraploid, fused cells were not frequently found in adult tissues.

To summarise, we show here that progeny of injected MEFs are found in organs from all three germ layers, mostly in the heart and brain (up to 30%), and are not tetraploid. 

#### 3.2.8. Preimplantation Development of Presumptive Embryonic–Somatic Chimaeras Obtained by Cumulus Cell Introduction

**Figure 10:**
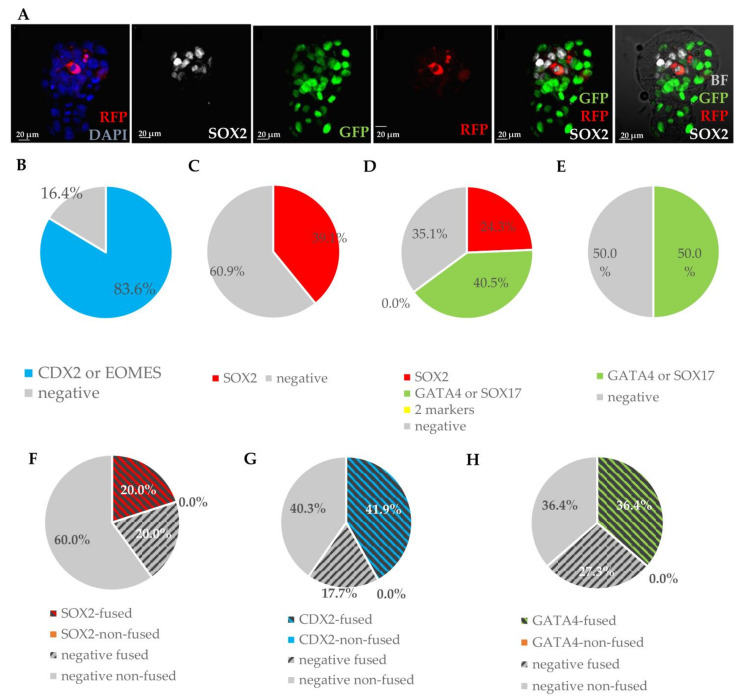
Analysis of marker expression in chimaeras with cumulus cells expressing RFP introduced to embryos. (**A**) Embryo with donor cumulus cell integrated with ICM and expressing SOX2 and GFP (cell fused with recipient GFP-expressing cell). (**B**) Cells stained for TE markers: positive for CDX2, EOMES (blue) and negative (grey). (**C**) Cells stained for EPI markers: positive for SOX2 (red) and negative (grey). (**D**) Cells stained for 2 ICM markers (for EPI and PrE marker): positive for SOX2 (red), GATA4, SOX17 (green) and negative (negative); 0.0% stands for the lack of double positive cells. (**E**) Cells stained for PrE markers: positive for GATA4, SOX17 (green) and negative (grey). (**F**) Cells stained for ICM markers: positive for SOX2 fused (red, striped), negative fused (grey, striped), negative, non-fused (grey). (**G**) Cells stained for TE markers: positive for CDX2, fused (blue, striped), negative fused (grey, striped), negative, non-fused (grey). (**H**) Cells stained for PrE markers: positive for GATA4, fused (green, striped), negative fused (grey, striped), negative, non-fused (grey).

To address the question of whether only foetal somatic cells can reprogram in the environment of the cleavage-stage embryo, we additionally analysed chimaeras produced by the introduction of adult somatic cells. RFP-positive cumulus cells were introduced into a total of 236 wild-type or GFP-positive E2.5 mouse embryos. Following 48-hour culture of these presumptive chimaeras, we found introduced cells (or progeny thereof) in 66.1% of the embryos at the blastocyst stage (n = 156). Chimaeric embryos were subsequently fixed and analysed by immunofluorescent staining for markers of first cell lineages: SOX2, OCT4 (Epi), GATA4, GATA6, SOX17 (PrE) and CDX2 or EOMES (TE) (Figure 10A). Many of the introduced cells expressed one of the lineage-specific markers, all in accordance with cell localisation, i.e., cells localised to TE expressed TE markers, and cells localised to the ICM expressed PrE or Epi markers. Overall, 83.6% of introduced cells localised to TE expressed TE-specific markers, 64.9% of introduced cells in ICM expressed EPI or PrE markers and co-expression of markers of different lineages was never observed (Figure 10 B–E). To analyse the possibility of the reprogramming of adult somatic cells by fusion with embryonic cells, we tracked marker expression in double transgenic presumptive chimaeras 48 h after cumulus cell introduction. This analysis revealed that chimaeric blastocysts contained both non-fused introduced cells, and cells apparently resulting from the fusion of embryonic and somatic cells (expressing both markers of introduced cells (RFP) and a marker of the recipient embryo (GFP)). However, none of the non-fused cells expressed any of the early-lineage markers (Figure 10 F–H). This result indicates that introduced cumulus cells are able to survive and to some extent integrate with the early embryo but, unlike foetal cells, can be reprogrammed only by fusion with embryonic cells.

## 4. Discussion

In this study, we investigated if somatic cells placed between blastomeres of 8-16-cell-stage recipient embryos can integrate and reprogram to a pluripotent state and to what extent they contribute to the formation of embryonic and adult tissues.

In an earlier study, we introduced three labelled MEFs to cleaving embryos [28]. After two days of culture, in 46.2% embryos, more than three MEFs were present, however, reprogramming of introduced cells was not analysed. Here, we show that MEFs introduced into the niche between blastomeres integrate with the early embryo at a high rate of 38%. Analyses of early-lineage marker expression showed that introduced cells exhibit TE, PrE or EPI-like transcriptional traits as early as the second day after introduction. The onset of marker expression of the blastocyst lineage that appears 48h after cell introduction suggests that the embryonic niche is a very effective reprogramming environment. Similar timing of reprogramming was observed when somatic cells were exposed to stem cell extract [17]. 

In our study, MEFs were reprogrammed by introduction into E2.5 embryos. Unlike mES cells, that can integrate with the embryo even when placed under the zona pellucida [48], MEFs must be placed in the middle of the embryo, among blastomeres, for successful integration. Even though many MEFs are excluded from the embryo, a high percentage of embryos (38%) incorporated at least one of the introduced MEFs. These cells mostly become part of the trophoblast, but they are also found in primitive endoderm and epiblast. 

It has been shown that hematopoietic stem cells introduced into blastocyst cavity can integrate with the embryo, and continue development as blood stem cells [27], i.e., along their mesenchymal lineage. Here, we show that it is possible to reprogram somatic cells in early embryo, such that they can survive long term in the embryo and are found in organs of all three germ layers: ectoderm (e.g., brain), endoderm (e.g., liver) and mesoderm (e.g., heart) of the developing embryo. MEFs have an advantage over adult hematopoetic stem cells in being at earlier stage of differentiation than the latter. This may explain the higher reprogramming diversity of MEFs as opposed to the other cells. The second factor in the comparison is time and the developmental stage of the recipient: MEFs are introduced to cleaving embryos and therefore have two more days to undergo reprogramming than do hematopoetic stem cells.The developmental potential of cells and their plasticity depend on their stage of development as well as the environment in which they are placed [49]. Blastomeres from 16-cell and 32-cell embryos are capable of retaining unrestricted developmental potential [50,51]. During blastocyst development, the potential of cells in the ICM is restricted, however, presumptive epiblast and primitive endoderm cells, when placed in E2.5 embryos [52], can transdifferentiate to a different cell lineage. 

Cells from early postimplantation embryos (E5.5) are able to contribute to chimaeras [53,54], reviewed recently by us [49]. MEFs obtained at E11.5–E13.5 are somatic cells of embryonal origin. Such cells, as well as precursor cells, can reprogram more easily than those originating from adult tissue [55,56,57,58]. In our experimental set-up, MEFs show reprogramming plasticity in the permissive environment of early embryos, possibly due to the innate expression of factors known to promote pluripotency, e.g., KLF4 [59,60,61]. It has been shown that in non-terminally differentiated cells, the induction of pluripotency requires fewer reprogramming factors than in original reprogramming experiments [62,63,64]. Cells of embryonic origin have a high proliferation potential, which is important for their ability to colonise the blastocyst [65,66].

It has been shown that MEFs can spontaneously fuse with ES cells and reprogram [14], which led us to the notion that fusion may be the possible mechanism of reprogramming. Previously, we hypothesised that fusion occurs on the 10th day of pregnancy as a mechanism of cell rescue [28]. However, the results presented here show that fusion occurs as early as during the first day after introduction. Experiments with GFP-positive recipient embryos and introduced RFP-positive MEFs confirmed that about 50% of MEFs are reprogrammed by cell fusion, as we observed the presence of double-labelled cells. When Oct4-GFP transgenic MEFs, at origin negative for pluripotency factor expression, were introduced into morula-stage embryos, the appearance of *Oct-4* promoter-directed EGFP expression was observed, which indicates quick reprogramming (see Figure S2). The timing of reprogramming by both mechanisms led us to the notion that this process resembles reprogramming by cell extract [67,68,69]. In our experiments, MEFs were not subjected to any pretreatment as opposed to the cells in cell extract studies. The environment of the early embryo creates a favourable niche for reprogramming. We have shown that markers of embryonic–somatic chimaerism can be detected at the DNA level as early as in egg cylinders. In our previous work, the failure to find a *lacZ* marker at this stage [28] can be attributed to the small number of *lacZ*-positive cells. The presence of DNA markers of chimaerism in normal E11.5 and retarded embryos, as well as in tissues of newborn and adult mice, proves the continuous persistence of introduced and reprogrammed cells in developing embryos. The presence of RFP-positive cells was detected in various organs originating from three germ layers, however, in gonads, the percentage of introduced cell progeny was very low, suggesting that reprogrammed MEFs were not capable of germline transmission.

In chimaeric foetuses, tetraploid cells were found more frequently than in the control. Ploidy analysis by FACS showed that both diploid and tetraploid RFP-positive cells are found in chimaeric foetuses and foetal membranes. These results are consistent with earlier data (e.g., [70]) that tetraploid cells can continue development in foetuses. 

However, in tissues of born animals, tetraploid cells’ contribution is reduced and comparable to the percentage of tetraploid cells in control mice. Tetraploid cells are the natural component of organs, such as the heart and liver, and are the product of incomplete cell divisions (liver) or cell fusion (heart muscle). Therefore, if tetraploid fused cells in chimaeric animals contribute to these organs, it would be impossible to differentiate these cells from cells originating from incomplete division. Markers of cells introduced to the embryo are still found in adult tissues. Tetraploid cells that were reprogrammed by fusion are found in foetuses and foetal membranes, but in born animals, a higher rate of tetraploidy was not confirmed. However, it is possible that cells reprogrammed by fusion predominantly localise to organs, where tetraploid cells naturally occur, such as in the heart and liver. 

Although tetraploid MEF progeny were found in significant numbers in foetal tissues, these cells were probably eliminated in adult animals. In contrast, the diploid progeny of MEFs were found both in foetal and adult tissues.

To address the question of whether only somatic cells of foetal origin can be reprogrammed to blastocyst lineages, we introduced adult somatic (cumulus) cells into cleaving embryos. Although the introduced cells expressed markers of blastocyst lineages, all of them appeared to have been fused with cells of the host embryo, suggesting that they can only be reprogrammed by cell fusion.

This result shows that adult (cumulus) cells are able to undergo reprogramming by fusion with embryonic cells, but may not be susceptible to reprogramming by the embryonic environment alone.

## 5. Conclusions

To summarise, we demonstrate that somatic cells are reprogrammed to blastocyst lineages in the unique niche of the preimplantation embryo. They can be reprogrammed both by fusion with blastomeres and without fusion, in the latter case remaining diploid. Surrounding blastomeres can create a favourable niche triggering the process of the reprogramming of somatic cells. 

Understanding how somatic cells are reprogrammed by contact with the niche of 8-16-cell embryos could create new insights into mechanisms of pluripotency induction, cell plasticity and development. 

## Data Availability

The data presented in this study are available on request from the corresponding author.

## Supplementary Materials

The following are available online at https://www.mdpi.com/2073-4409/10/3/490/s1, Table S1: Contribution of donor cells to blastocyst lineages and expression of blastocyst markers; Table S2: Number of foetuses and their developmental status; Table S3: Contribution of males to experimental foetuses; Table S4: Donor contribution to chimaeras between donor (ROSA26-lacZ) MEFs and recipient (DBA/2) embryos; Table S5: Donor markers in foetal and foetal membrane samples sorted into diploid and tetraploid fractions; Table S6: Donor markers’ contribution to organs of born animals; Figure S1: Expression of *Oct 4* and *Nanog* pluripotency factors in MEF-RFP; Figure S2: Development of chimaeric embryo with transgenic MEF cells introduced to 8-16-cell embryo.
