# Supplementary material for: Embryonic Environmental Niche Reprograms Somatic Cells to Express Pluripotency Markers and Participate in Adult Chimaeras
Source: Cells. 2021 Feb 25;10(3):490. doi: 10.3390/cells10030490 (PMC7996319; doi:10.3390/cells10030490)
Supplement: Supplementary file 1 [file cells-10-00490-s001.pdf]

Table S1. Contribution of donor cells to blastocyst lineages and expression of blastocyst markers.

	Cells in TE	Cells in ICM					
**Number of cells in layer**	**69 (32 blastocysts)**	**72 (41 blastocysts)**					
**Cells were stained for marker:**	**Cdx2**	**Nanog**	**Gata4**	**N+G4**			**Fused (Pdgfr+)**
**Number of cells stained for**	**60**	**25**	**6**	**31**			**21**
**Expression of marker in each cell**	**Cdx2**	**Nanog**	**Gata4**	**Nanog**	**Gata4**	**Nanog and Gata4**	**Pdgfr**
**Number of cells**	**38 (24 blastocysts)**	**15 (13 bl)**	**6 (6 bl)**	**12 (9 bl)**	**9 (6 bl)**	**4 (2 blastocysts)**	**10 (9 blastocysts)**
**% positive**	**63%**	**60%**	**100%**	**81%**			

Table S2. Number of foetuses and their developmental status.

	Normal	Retarded 0.5-2 days	Retarded	Large egg cylinder	Degenerating embryos
**Foetuses from ED 13.5**	**14**	**0**	**0**	**0**	**3**
	**82.4%**	**0.0%**	**0.0%**	**0.0%**	**17.6%**
**Foetuses from ED 10.5-12.5**	**11**	**17**	**4**	**3**	**3**
	**28.9%**	**44.7%**	**10.5%**	**7.9%**	**7.9%**
**SUM**	**25**	**17**	**4**	**3**	**6**
	**45.5%**	**30.9%**	**7.3%**	**5.5%**	**10.9%**

Table S3. Contribution of males to experimental foetuses.

	Normal	Slightly retarded	Retarded	Degenerating
**Sry^+**	**6**	**1**	**2**	**2**
**total**	**7**	**2**	**4**	**4**

Table S4. Donor contribution to chimaeras between donor (ROSA26-lacZ) MEFs and recipient (DBA/2) embryos.

Stage of the foetus	Samples from	Presence of donor ROSA26-lacZ markers				
		**No of samples**	***LacZ*** **+**	***D3Mit200*** **MS +**	**Both**	**Positive**
**Normal**	**Foetus**	**7**	**4**	**1**	**2**	**7/7 (100%)**
	**ExEm**	**7**	**4**	**3**	**0**	**7/7 (100%)**
**Retarded 0.5-2 days**	**Foetus**	**3**	**2**	**0**	**1**	**3/3 (100%)**
	**ExEm**	**3**	**1**	**1**	**0**	**2/3 (67%)**
**Retarded**	**Foetus**	**3**	**2**	**1**	**0**	**3/3 (100%)**
	**ExEm**	**2**	**1**	**0**	**0**	**1/2 (50%)**
**Other***	**Other****	**6**	**1**	**3**	**0**	**4/6 (67%)**
**Total**	**Foetuses**	**19*****	**9**	**5**	**3**	**17 (89%)**

**ExEm**	**12**	**6**	**4**	**0**	**10 (83%)**
**Total**	**31**	**15**	**9**	**3**	**27 (87%)**

ExEm - Extraembryonic tissues

*** Implantation sites/degenerating embryos**

**all material found in implantation sites possibly containing degenerating embryo

*** thirteen foetuses plus 6 "others"

Table S5. Donor markers in foetal and foetal membrane samples sorted into diploid and tetraploid fractions.

M	Marker	Foetal/extraembryonic	Ploidy	*LacZ*	*D3Mit200*
1	Foetus 1	foetal tissues	4n		
2		foetal tissues	2n		
3	Foetus 2	foetal tissues	4n	+	+
4		foetal tissues	2n	+	+
5	Foetus 3	yolk sac	4n	+	+
6		yolk sac	2n		
7	ROSA donor	foetal tissues	2n	+	+
8	DBA/2	foetal tissues	2n		
M	marker				

Table S6. Donor markers' contribution to organs of born animals.

	lacZ					*D3Mit200*				
	♂ 1	♂ 2	♂ 3	♂ 4	♂ 5	♀ 6	♀ 7	♂ 8*	♂ 9	♂ 10
**brain**	+	-	-	+	+	+	+	-	-	**no sample**
**gonad**	+	+	-	+	-	-	-	-	-	**no sample**
**heart**	+	+	-	+	+	-	-	-	+	-
**intestine**	+	+	+	+	+	-	-	-	-	-
**kidney**	-	+	+	+	-	-	-	+	+	**no sample**
**liver**	+	-	-	+	+	+	-	+	-	-
**lung**	+	-	-	+	-	-	-	+	+	-
**muscle**	+	-	-	+	+	-	-	-	-	**no sample**
**skin**	+	+	+	+	-	-	-	-	-	-
**spleen**	-	-	+	-	+	-	-	+	-	**no sample**
**tumor**		**no sample**			+				**no sample**	
**Positive samples**	**8**	**5**	**4**	**8**	**7**	**2**	**1**	**4**	**3**	**0**
**No of samples**	**10**	**10**	**10**	**10**	**11**	**10**	**10**	**10**	**10**	**5**

*this male died at 1 month old of unknown reason, however samples were collected soon after death and taken for analyses

Figure S1. Expression of *Oct 4* and *Nanog* pluripotency factors in MEF-RFP.

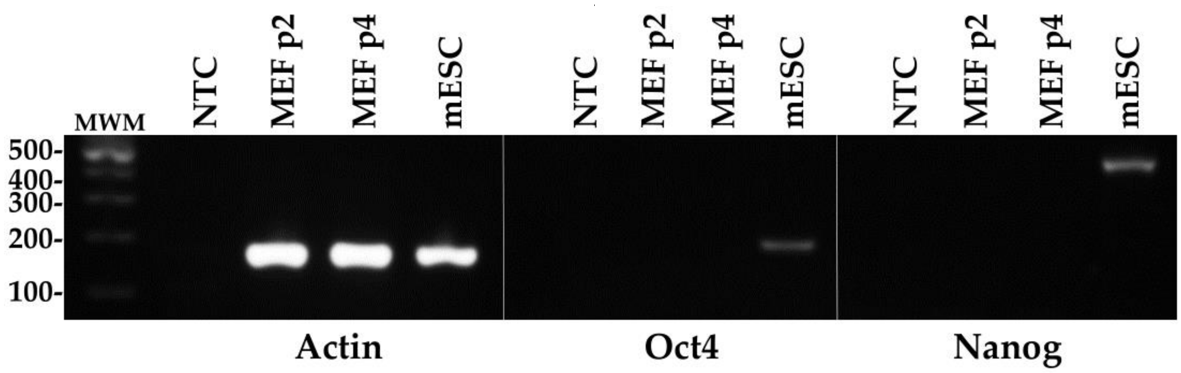

Figure S2. (A) Development of chimaeric embryo with transgenic MEF cells introduced to 8-16-cell embryo. (B) Table showing number of embryos in which onset of OCT4-GFP expression was observed. *StartPoint is an observation directly after manipulations, which for a particular embryo may be up to 6 h after manipulation.

A

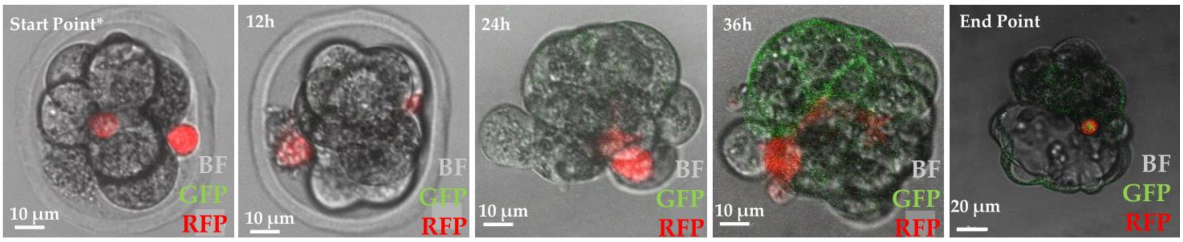

B

	Start Point*	12h	24h	36h	End Point
**Number of embryos with cells expressing OCT4-GFP**	5	13	18	23	25
**Number of embryos with onset of OCT4-GFP expression**	5	8	5	5	2
